# Patient-Reported Outcomes Following Two-Tined Three-Site Conventional Radiofrequency Ablation of Genicular Nerves for Chronic Knee Pain

**DOI:** 10.7759/cureus.116510

**Published:** 2026-09-18

**Authors:** Mingda Chen, Sercan Tosun, Nicolas R Thompson, Kush Goyal

**Affiliations:** 1 School of Medicine, Case Western Reserve University, Cleveland, USA; 2 Imaging Institute, Cleveland Clinic, Cleveland, USA; 3 Department of Quantitative Health Sciences, Cleveland Clinic, Cleveland, USA; 4 Center for Spine and Pain, Cleveland Clinic, Cleveland, USA

**Keywords:** chronic pain, classical three-site, genicular nerves, knee osteoarthritis, patient-reported outcomes, radiofrequency ablation

## Abstract

Background: Genicular nerve radiofrequency ablation (GNRFA) has emerged as an effective therapy for chronic knee pain. Although some studies have evaluated its effectiveness, there is limited real-world data on the long-term patient-reported outcome (PRO) data for GNRFAs.

Objective: This study aimed to evaluate PROs following conventional GNRFAs performed using two-tined electrodes and the classical three-site technique targeting the superior medial, superior lateral, and inferior medial genicular nerves.

Methods: We performed a retrospective chart review to gather data from consecutive GNRFAs at an academic medical center from 2016 to 2023. Inclusion criteria were all adult patients who received a conventional GNRFA performed using the classical three-site technique using two-tined electrodes. All included subjects were confirmed to have a positive diagnostic response (≥50% Numeric Rating Scale (NRS) reduction) to a previous genicular nerve block. Primary outcomes were NRS pain scores, Patient Reported Outcomes Measurement Information System (PROMIS) Global Health, and Patient Health Questionnaire-9 (PHQ-9).

Results: A total of 31 patients underwent 38 GNRFAs. Relative to the pre-procedure baseline, the average NRS reduction post-procedurally was -3.17 during 0-3 months (p<0.0001), -1.31 during 3-6 months (p<0.05), and -1.92 during 6-12 months (p<0.05). Half of all patients maintained pain relief (NRS reduction greater than or equal to a minimal clinically important difference (MCID) of -2.0) for 11.17 months (95% CI: 7.29, 13.40). Although there was a positive trend for improvement in the quality-of-life measures, no statistically significant post-procedure improvement or worsening was observed during all time periods for PROMIS Physical Health, PROMIS Mental Health, and PHQ-9. Lastly, a history of depression was associated with treatment failure.

Conclusion: In this real-world cohort, conventional GNRFA using the classical three-site technique demonstrated robust short-term pain reduction, and at least half of the cohort maintained clinically meaningful pain improvements for 11 months. For chronic knee pain, GNRFA is a reasonable therapeutic option in appropriately selected patients.

## Introduction

Genicular nerve interventions such as genicular nerve block (GNB) and genicular nerve radiofrequency ablation (GNRFA) have emerged as meaningful treatment options for painful knee osteoarthritis (OA) [1]. Patients who receive a genicular nerve intervention usually present with persistent knee pain despite conservative management but have not yet received a total knee arthroplasty (TKA) or experienced post-TKA knee pain that is refractory to conservative management options. Both GNB and GNRFA are minimally invasive interventions that target genicular nerves to achieve sensory denervation of the knee joint capsule [2-5]. A GNB uses a local anesthetic with or without corticosteroid to numb the genicular nerves [6], while a GNRFA uses traditional or cooled radiofrequency ablation to create localized lesions to inactivate the genicular nerves [1].

Classically, GNRFA procedures are performed using a three-site technique to target the superior medial genicular nerve (SMGN), superior lateral genicular nerve (SLGM), and inferior medial genicular nerve (IMGN) [6,7]. In recent years, innovative approaches have targeted additional sensory nerves such as the infrapatellar branch of the saphenous nerve, the recurrent fibular nerve, and the nerves to the vastus medialis, vastus intermedius, and vastus lateralis muscles [1,8-13]. Currently, there is ongoing debate over the ideal selection of genicular nerve targets as well as patient selection criteria for these procedures.

Over the past decade, increasing evidence has supported the efficacy and effectiveness of GNRFA. Although multiple GNRFA trials have been performed in controlled clinical settings, there have been a limited number of retrospective studies that examined the long-term patient-reported outcome (PRO) data (e.g., 8-12+ months post-procedure) for GNRFAs in real-world clinical practice. Additionally, there remains significant variation in insurance coverage for GNRFA, where many insurance companies still do not cover GNRFA despite published evidence supporting its effectiveness at relieving chronic knee pain. Despite recent studies that highlighted the effectiveness of GNRFAs [14-17], there is still a need for data on the long-term therapeutic effect (i.e., 6 months to >1 year) of GNRFAs.

Across a study period of eight years, the aim of this study was to gather a real-world cohort of chronic knee patients treated by conventional GNRFA with long-term follow-up. We focused on procedures performed using the classical three-site radiofrequency ablation approach, the predominant technique at our institution. The primary outcomes were patient-reported pain scores and quality-of-life questionnaires collected at office visits. The three main PROs of interest were the Numeric Rating Scale (NRS) for knee pain levels, the Patient Health Questionnaire-9 (PHQ-9) for depression severity rating, and the Patient Reported Outcomes Measurement Information System (PROMIS) Global Health questionnaire.

## Materials and methods

Overview and study design

We performed a retrospective review of the electronic medical records to identify consecutive GNRFA procedures performed from January 2016 to December 2023 at Cleveland Clinic, a large academic tertiary care center in Cleveland, Ohio, by a single fellowship-trained physician. All procedures were performed with a two-tined electrode system (single burn at each of the three sites). Ethical approval was obtained from the Cleveland Clinic Institutional Review Board (approval number: 23-734) prior to study initiation.

Procedure technique

The patient's knee was flexed with a bolster support to optimize the view of the knee joint space, and then the knee was prepped/draped in a sterile fashion. Three skin wheals were raised using 1% preservative-free lidocaine (0.5 mL per site) near the SMGN, SLGN, and IMGN based on bony landmarks. In the anteroposterior (AP) plane with the patella centered, a skin wheal was placed for local anesthesia. Next, 20-gauge, 100-mm two-tined radiofrequency rhizotomy needles were introduced and advanced under direct fluoroscopic visualization in the AP and lateral planes. The distal tip of the needle was advanced across ~60-90% of the femoral or tibial shaft on lateral view, staying 1-2 mm superficial to the periosteum in the AP view (Figure 1). The SMGN target was the confluence of the medial femoral shaft and the medial epicondyle. The SLGN target was the confluence of the lateral femoral shaft and lateral femoral condyle. The IMGN target was the confluence of the medial tibial shaft and tibial flare. After proper needle placement was confirmed, sensory and motor stimulation was performed prior to lesioning. Using the Stryker MultiGen radiofrequency generator (Kalamazoo, Michigan, United States), single lesioning was performed at 80°C for 90 seconds at each of the three sites simultaneously. After lesioning, we administered 0.75% preservative-free bupivacaine at each site (0.5 mL at each site).

**Figure 1:**
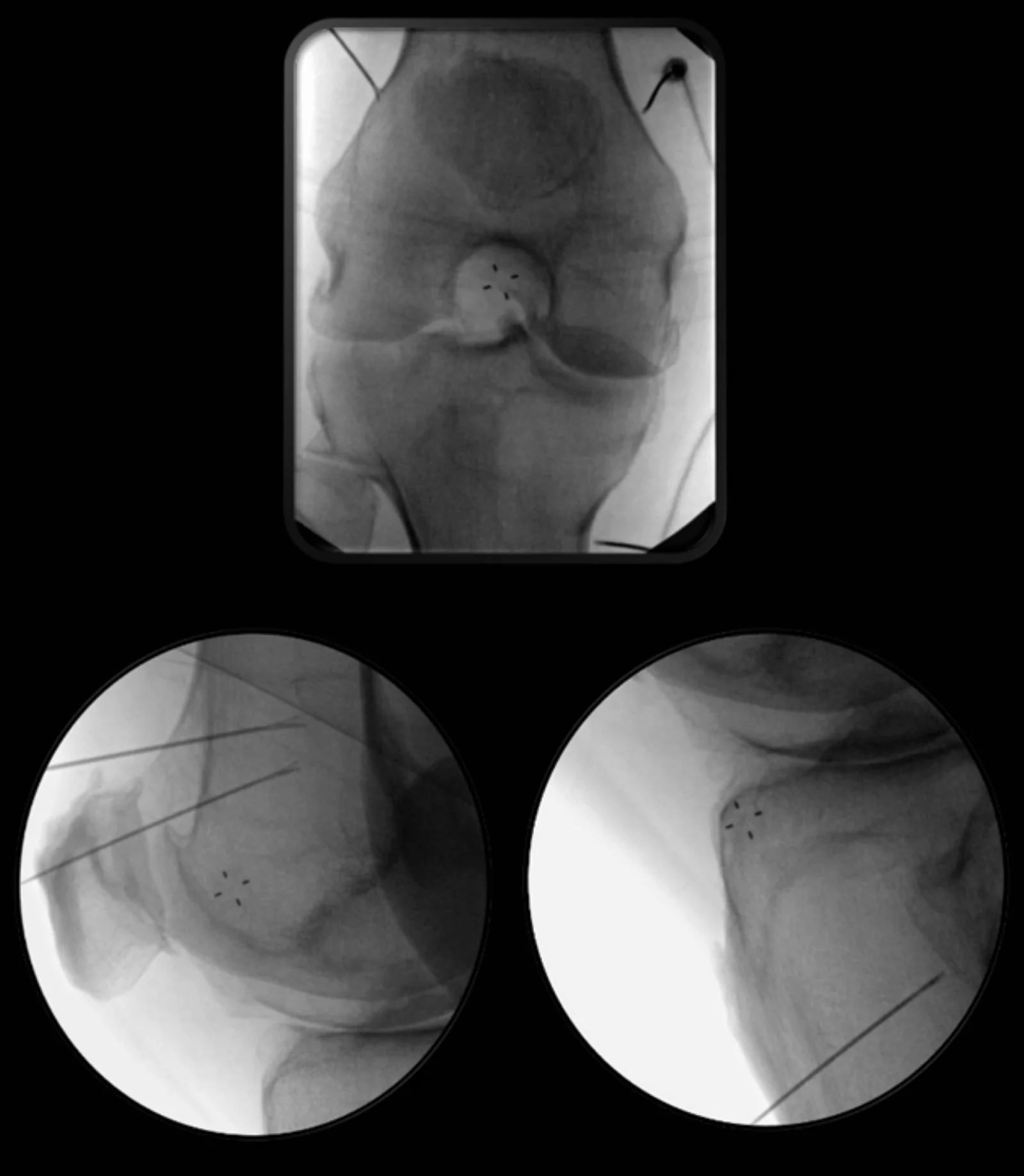
Anteroposterior (top) and lateral knee radiographs (bottom) showing the classical three-site genicular nerve radiofrequency ablation approach using two-tined electrodes, targeting the superior medial, superior lateral, and inferior medial genicular nerves

Study population

We gathered consecutive patients (aged 18 years or older) who underwent a GNRFA for painful knee OA during the study period. Study inclusion criteria required a positive diagnostic response (≥50% NRS reduction) to a diagnostic GNB prior to their GNRFA. Patients with a Kellgren-Lawrence (KL) grade less than 2 were excluded because they did not have definitive knee OA [18,19]. We also excluded those who did not follow up post-procedurally. KL grading was performed on weight-bearing knee radiographs by two reviewers (blinded to outcomes data). We also excluded GNRFAs performed using modified approaches (i.e., expanded protocols, cooled/bipolar radiofrequency ablation) to avoid bias from procedure/technique heterogeneity and due to the inadequate sample size. We manually reviewed patient charts, radiology reports, prior medical imaging, and intra-procedural fluoroscopy images to ensure adherence to the eligibility criteria.

Data collection

We performed a manual chart review to the collected data on the following pre-procedural variables: basic demographic information, relevant comorbidities including depression, anxiety disorder, fibromyalgia, chronic pain syndrome, meniscal tear (confirmed on magnetic resonance imaging (MRI)), diabetes, alcohol abuse, and gout, smoking status, chronic corticosteroid use, and prescription opioid usage (quantified by morphine milligram equivalents (MME)). We also gathered knee OA characteristics (duration of knee pain prior to procedure and symptom laterality) and a history of knee OA treatments (intra-articular steroid injections, visco-supplementation, GNBs, lower extremity surgery, and physical therapy). All variables were manually collected and verified based on the information from clinical assessment notes, procedural notes, radiology reports, medication dispense reports, and problem lists.

Data were collected on the following periprocedural variables: American Society of Anesthesiologists (ASA) classification, sedative use, procedure laterality, KL grade at procedure, and pre-procedural NRS scores. At post-procedure follow-up visits, we collected data on PRO measures, utilization of adjuvant treatments (number of knee OA physical therapy sessions), complications, and interval knee surgery. We used existing validated and standardized questionnaires for collecting all PROs, including the NRS pain scores, PROMIS Global Health 10-item questionnaire (includes Physical Health (PH) and Mental Health (MH) components) [20], and PHQ-9 [21]. We only used pre-procedure PRO scores within three months of the procedure date because utilization of older scores may not represent an accurate baseline for comparison.

Statistical analysis

For GNRFA outcomes, all PRO scores were grouped into the following post-procedure follow-up periods: 1-90 days (0-3 months), 91-182 days (3-6 months), 183-365 days (6-12 months), and 366+ days. We used the Wilcoxon signed-rank test for paired comparisons between pre-procedure and post-procedure PRO scores. We selected the minimal clinically important difference (MCID) of -2.0 for NRS rating of knee pain as previously reported in the literature [22-24]. We defined a non-response subgroup and a corresponding positive response subgroup to compare potential factors associated with poor treatment response. Non-response (not clinically significant) was defined as <50% NRS score reduction [24] or <75% patient-reported pain relief within three months post-procedurally. We used Welch's t-test (assuming unequal variance) to evaluate potential factors suspected to be associated with non-response to treatment. Analyses were performed at the procedure level. Multiple comparisons were corrected by the Bonferroni approach when applicable. All tests were two-sided. We used available-case analysis to handle all missing data. All data analyses were performed in R Version 4.3.1 (R Foundation for Statistical Computing, Vienna, Austria).

## Results

Demographic and procedure characteristics

A total of 38 GNRFAs (45 knees) from 31 patients were eligible for analysis during the study period. The average age in this study was 66.1±12.8 years, with a majority of female patients (61.3%) (Table 1). Most patients were obese (22/31, 71% BMI ≥30) with an average BMI of 35.60 (SD 9.71). The patients were from diverse backgrounds (51.6% White, 32.3% Black, 12.9% multi-racial or other, and 3.2% Asian), and 16.1% Hispanic. The majority of patients were referred by orthopedic surgery/sports medicine (35.48%, 11/31), spine medicine/physical medicine and rehabilitation (19.35%, 6/31), or neurology/neurosurgery (16.13%, 5/31).

**Table 1:** Demographic and baseline clinical characteristics of all genicular nerve radiofrequency ablation patients Data have been represented as N (%) and mean±SD.

n	31
Sex (%)	
Female	19 (61.29%)
Male	12 (38.71%)
Age, mean (SD)	66.13 (±12.76)
Body mass index, mean (SD)	35.60 (±9.71)
Race (%)	
White	16 (51.61%)
Black	10 (32.26%)
Other/multi-racial	4 (12.90%)
Asian/Pacific Islander	1 (3.23%)
Ethnicity (%)	
Non-Hispanic	26 (83.87%)
Hispanic	5 (16.13%)
Diabetic (%)	16 (51.61%)
Smoking status (%)	
Current smoker	3 (9.68%)
Ever smoker (quit smoking for at least 1 year)	13 (41.94%)
Never smoker	15 (48.39%)
History of meniscal tear (%)	
None	18 (58.06%)
Ipsilateral	9 (29.03%)
Bilateral	2 (6.45%)
Contralateral	2 (6.45%)
History of fibromyalgia (%)	3 (9.68%)
History of chronic pain syndrome (%)	7 (22.58%)
History of anxiety disorder (%)	13 (41.94%)
History of depression (%)	14 (45.16%)
History of alcohol abuse (%)	2 (6.45%)
History of gout or pseudogout (%)	5 (16.13%)
History of chronic corticosteroid use (%)	2 (6.45%)
Prescription opioid use (%)	
Current	5 (16.13%)
Daily morphine milligram equivalents, mean (SD)	24.50 (±5.12)
Prior opioid use but stopped	4 (12.90%)
Never	22 (70.97%)
Laterality of knee osteoarthritis (%)	
Bilateral	22 (70.97%)
Left	4 (12.90%)
Right	5 (16.13%)
Duration of knee pain, years, mean (SD)	5.64 (±4.16)
Physical therapy use before procedure (%)	20 (64.52%)
Number of physical therapy sessions within 1 year, mean (SD)	4 (±5.14)
Number of prior intra-articular knee steroid injections (%)	
None or unknown	8 (25.80%)
1	15 (48.39%)
2	5 (16.13%)
≥3	3 (9.68%)
Number of prior knee visco-supplement treatments (%)	
0	19 (61.29%)
1	12 (38.71%)
Number of prior genicular nerve blocks (%)	
1	25 (80.64%)
2	5 (16.13%)
≥3	1 (3.23%)
History of prior knee surgery (%)	16 (51.61%)
Type of prior knee surgery	
Knee arthroscopy with meniscectomy or meniscal repair	11 (68.75%)
Knee arthroplasty	5 (31.25%)
History of prior hip surgery (%)	4 (12.90%)
History of prior ankle foot surgery (%)	1 (3.23%)
Referral specialty (%)	
Orthopedic surgery/sports medicine	11 (35.48%)
Spine medicine/physical medicine and rehabilitation	6 (19.35%)
Neurology/neurosurgery	5 (16.13%)
Primary care	3 (9.68%)
Other	3 (9.68%)
No referral	3 (9.68%)

Of the GNRFAs performed, 81.6% (31/38) were first-time procedures, and 18.4% (7/31) were repeat procedures. A small proportion of patients (5/31, 16.1%) reported active prescription opioid use at the time of their procedure, with an average daily opioid use of 24.50±5.12 MME/day. Before their first GNRFA, the majority of patients had an intra-articular corticosteroid knee injection (23/31, 74.2%), and some also had visco-supplementation (12/31, 38.7%).

Pre-procedurally, the average duration of knee pain was 5.64 (SD 4.16) years. The majority of patients (63%) had severe knee OA (KL grade 4) at the time of procedure. There were five patients (16.1%) with a history of TKA prior to their first GNRFA, and the subgroup comparison did not show statistically significant outcome differences comparing the post-TKA versus native knees (p>0.05). Post-procedurally, only 21% of patients went to their prescribed post-intervention physical therapy (Table 2). Overall, the average duration of follow-up in this sample was 11.39 months (SD 9.84). We did not observe any adverse events or complications related to the GNRFA procedure in this cohort.

**Table 2:** Procedure characteristics of genicular nerve radiofrequency ablation Data have been represented as N (%) and mean±SD.

n	38
Procedure laterality (%)	
Bilateral	7 (18.42%)
Unilateral	
Left	17 (44.74%)
Right	14 (36.84%)
American Society of Anesthesiologists Physical Status classification (%)	
ASA II	12 (31.58%)
ASA III	21 (55.26%)
ASA IV	5 (13.16%)
Sedation performed (%)	28 (73.68%)
Sedative type, may be a combination (%)	
PO clonazepam	0 (0%)
PO diphenhydramine	0 (0%)
IV midazolam	23 (60.53%)
IV fentanyl	22 (57.89%)
None	10 (26.32%)
Kellgren-Lawrence grade at procedure (%)	
Grade 2 (minimal)	4 (12.90%)
Grade 3 (moderate)	7 (22.58%)
Grade 4 (severe)	20 (64.52%)
Numeric Rating Scale pain score pre-procedure, mean (SD)	6.87 (±1.77)
Immediate pain relief, mean percentage (SD)	85.53 (±21.37)
Immediate pain relief groups (%)	
No relief (0%)	1 (2.63%)
Minimal-mild relief (1-49%)	1 (2.63%)
Moderate relief (50-74%)	2 (5.26%)
Significant relief (75-100%)	34 (89.47%)
Amount of physical therapy after procedure	
0 sessions	30 (78.95%)
1-5 sessions	6 (15.79%)
6-10 sessions	2 (5.26%)

GNRFA outcomes

In paired comparisons to the pre-procedure baseline, the mean NRS score reduction post-procedurally was -3.17 during 0-3 months (p<0.0001), -1.31 during 3-6 months (p<0.05), and -1.92 during 6-12 months (p<0.05) (Table 3; Figure 2). There was no significant difference from baseline after one year post-procedurally. Subjectively, the mean patient-reported percent relief following their GNRFA was 81.6%. Although there was a positive trend for improvement in the quality-of-life measures, no statistically significant post-procedure improvement or worsening was observed during all time periods for PROMIS-PH, PROMIS-MH, and PHQ-9 (p>0.05; Table 3).

**Table 3:** Change in patient-reported outcome measures (post-procedure minus pre-procedure scores) for NRS, PROMIS-PH, PROMIS-MH, and PHQ-9 for patients who underwent a genicular nerve radiofrequency ablation Data have been represented as N and mean±SD. A p-value of <0.05 was considered statistically significant. NRS: Numeric Rating Scale; PROMIS: Patient Reported Outcomes Measurement Information System; PH: Physical Health; MH: Mental Health; PHQ-9: Patient Health Questionnaire-9

	n	Baseline	1-90 days	91-182 days	183-365 days	366+ days
Δ NRS, mean (SD)	38	6.87 (±1.77)	-3.17 (±2.56)	-1.31 (±2.27)	-1.92 (±3.09)	-0.54 (±2.98)
Δ PROMIS-PH, mean (SD)	36	37.22 (±7.33)	+1.13 (±2.39)	+1.21 (±3.37)	+0.07 (±3.39)	+1.81 (±3.18)
Δ PROMIS-MH, mean (SD)	35	40.57 (±8.81)	+0.91 (±6.82)	-0.21 (±2.85)	+3.42 (±6.17)	+3.16 (±10.23)
Δ PHQ-9, mean (SD)	37	7.48 (±5.81)	+0.78 (±7.86)	+0.60 (±5.14)	+1.42 (±6.56)	+0.67 (±5.31)

**Figure 2:**
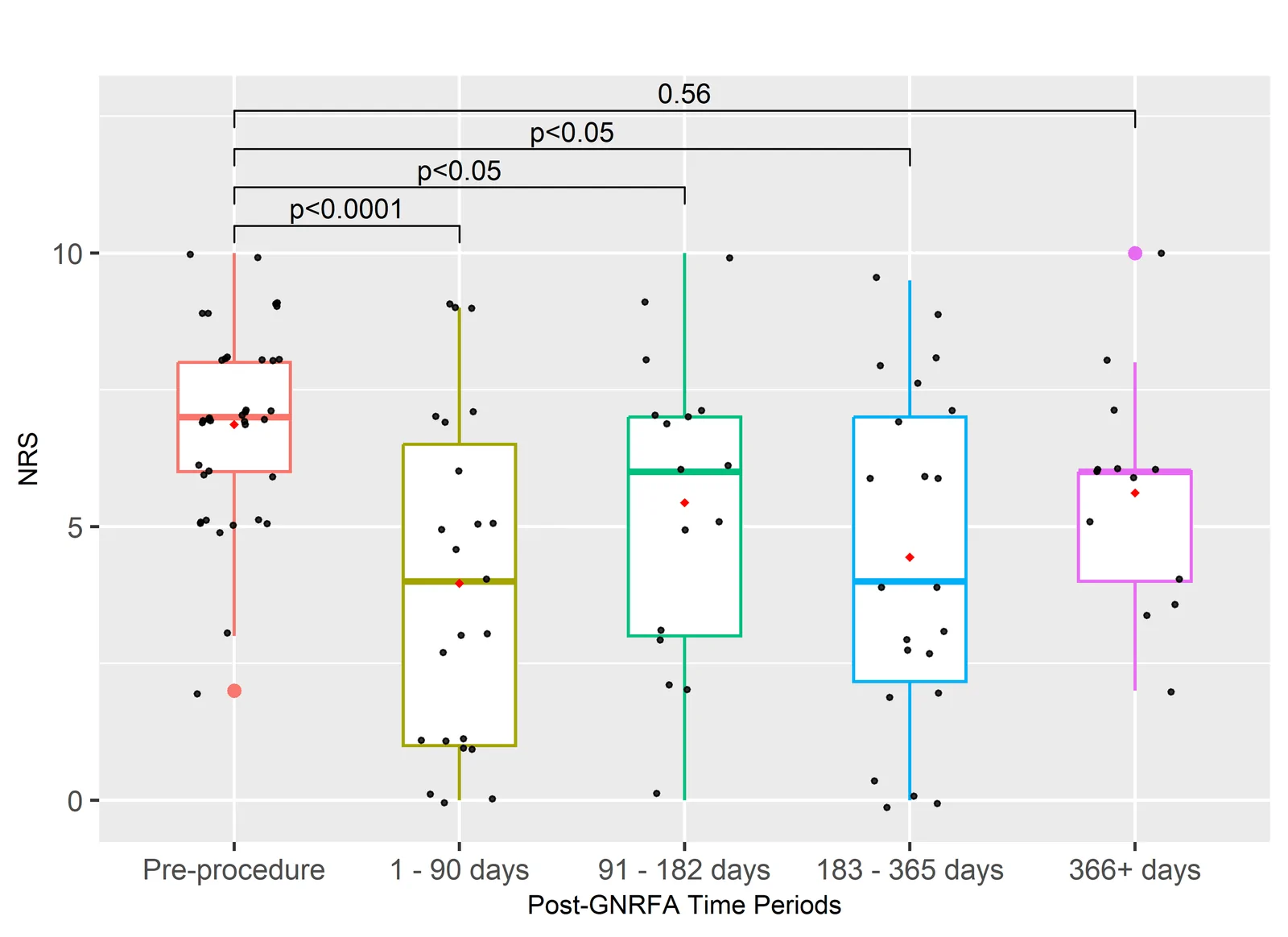
Comparisons of baseline versus post-procedure NRS scores from patients who underwent a classical three-site GNRFA, stratified by follow-up periods Boxplots of NRS pain scores during each follow-up period. A p-value of <0.05 was considered statistically significant. NRS: Numeric Rating Scale; GNRFA: genicular nerve radiofrequency ablation

After their GNRFA procedure, 50% of patients maintained a NRS reduction greater than or equal to the MCID of -2.0 for at least 11.17 months (95% CI: 7.29, 13.40) (Figure 3). A history of depression was associated with non-response (n=12 GNRFAs; p=0.01944). Of note, the following factors were not significantly associated with treatment failure (p>0.05): BMI, KL grade, physical therapy adherence, fibromyalgia, chronic pain syndrome, pre-procedural opioid usage, and prior knee surgery. Lastly, nearly a quarter of all patients (22.6%, 7/31) underwent a TKA after their GNRFA, with an average time to TKA of 25.2 months.

**Figure 3:**
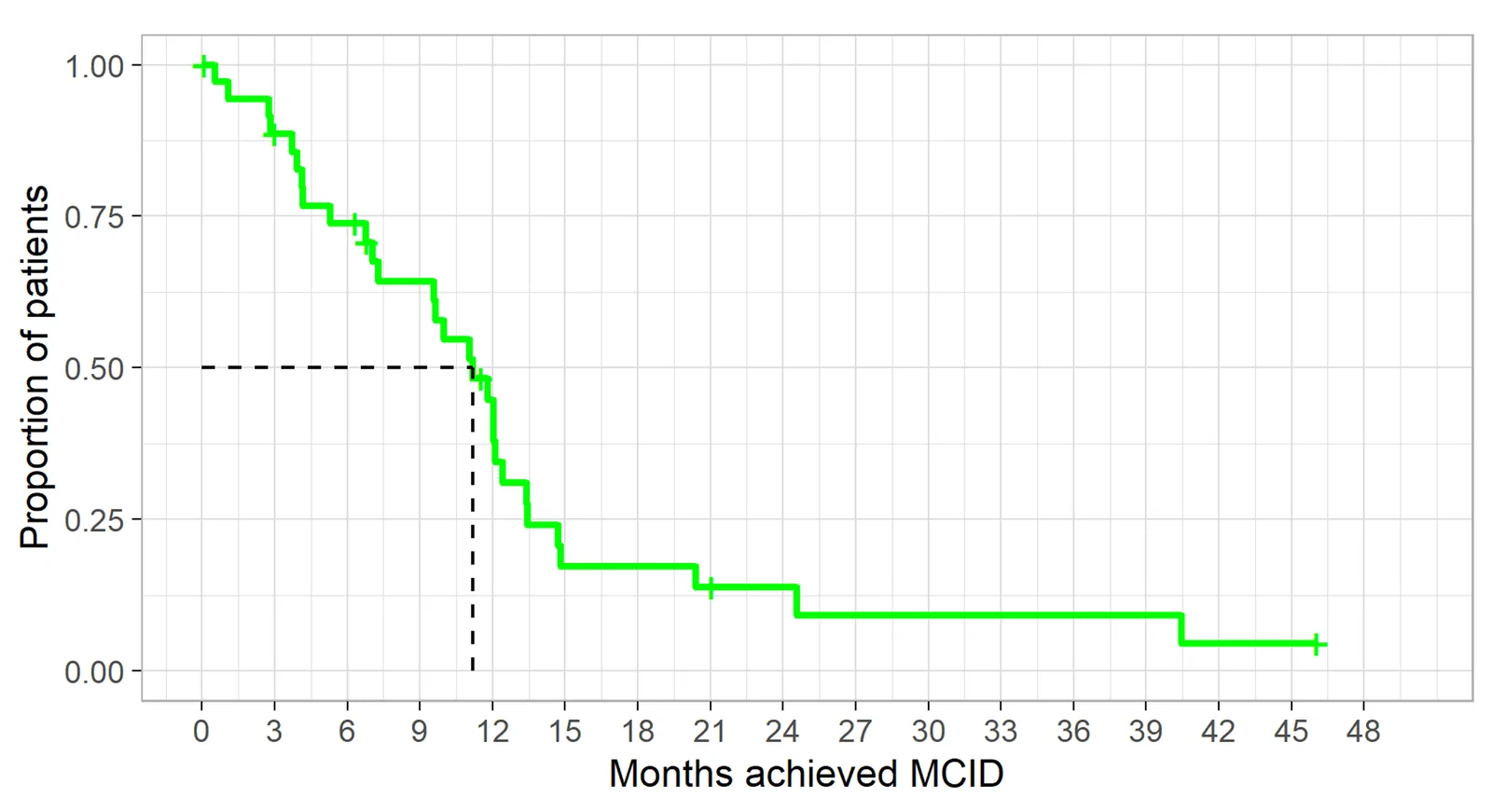
Survival curve portraying the number of months that patients sustained the MCID of -2.0 for NRS reduction after a GNRFA Data have been represented as proportion of patients (ranging from 1.0 to 0.0). NRS: Numeric Rating Scale; MCID: minimal clinically important difference; GNRFA: genicular nerve radiofrequency ablation

## Discussion

In this study, we successfully gathered long-term follow-up data on a real-world cohort of chronic knee patients treated by conventional GNRFA performed using the classical three-site approach. In the context of existing literature, our findings are consistent with existing evidence on short-term and long-term GNRFA outcomes. Overall, we found both statistically and clinically significant reductions in NRS pain scores during the 0-3-month follow-up period with some enduring pain relief that lasted 3-12 months post-procedurally.

The magnitude of NRS reduction in this study is similar to multiple GNRFA studies during the 1-3-month follow-up period and diminished after three months post-procedure and beyond [2,25-27]. The findings of this GNRFA study are consistent with existing literature [28], although we only observed minor improvements (not statistically significant) in PROMIS Global Health scores post-procedurally [17]. In terms of the magnitude of NRS reduction, our results showed slightly greater pain reduction than some studies [5,29-32] and slightly lower than others [25-27,33-35]. The slight variations in the magnitude of the NRS changes can be attributed to procedure techniques and case-mix complexity. Compared to our study, the NRS pain reductions reported by cooled GNRFA studies and expanded protocols were slightly higher and more enduring [9,13,36-41] although not indicative of superiority. At our practice, expanded protocol GNRFAs were occasionally offered (when appropriate) to patients who achieved some but <6 months of substantial relief from their classical three-site GNRFA.

Compared to existing GNRFA studies, a similar proportion of our patients underwent TKA post-procedurally [39], with a mean time to TKA of >2 years after their initial procedure. Based on the results of the post-TKA knee subgroup analysis, we found no significant outcome differences between the native knee subgroup and post-TKA knee subgroup, supporting existing evidence that GNRFA can be effective for post-TKA knee pain [16,42].

A history of depression was associated with non-response to GNRFA. Depression can influence knee pain [43] and is a predictor of persistent pain after TKA [44]. Patients with a history of depression responded worse to GNRFA than those without depression, which is consistent with other reports in the literature [24,45,46]. Taken together, GNRFA patients with pre-existing depression may benefit from pre-/post-procedural mental health treatment and participation in a multidisciplinary pain program or functional restoration program [47,48].

Lastly, our patient cohort is a complex population with a high burden of comorbidities because many were previously treated and referred by orthopedic or sports medicine practitioners. An interventional spine practice often manages knee OA in the context of additional comorbidities such as spine pathology, chronic pain syndrome, fibromyalgia, anxiety, and depression. In contrast, a predominantly orthopedic or sports medicine practice more often encounters patients with isolated knee pathology and fewer overlapping chronic pain or psychosocial conditions. This distinction highlights the greater clinical complexity and multifactorial background of our patient population.

Strengths and limitations

This study's main strength is its long study period (eight years) and the use of multiple different PRO measures (NRS, PROMIS-PH, PROMIS-MH, PHQ-9) to examine GNRFA outcomes. This real-world cohort consisted of many patients who often presented to our institution with late-stage knee OA (based on KL grade), who were previously treated by other orthopedic, sports medicine, and physical medicine and rehabilitation providers. One limitation is our sample size of GNRFAs, which is limited to a single provider's clinical practice. Although we performed linear mixed model analyses in an attempt to account for repeated measures and potential confounding (such as age, sex, BMI, and opioid use), the mixed model results (not shown due to being less appropriate given the limited sample size) were not vastly different than results from the unadjusted models. A limitation of our institution's electronic records is the lack of other knee outcome measures, such as the Knee Injury and Osteoarthritis Outcome Score (KOOS) and Western Ontario and McMaster Universities Osteoarthritis Index (WOMAC). Despite gathering long-term follow-up information for many patients, we observed that a sizable portion of patients were lost to follow-up after six months post-procedure or followed up at another institution. Because data collection was limited to our own institution's health records, we may have missed patient history and follow-up information from other health systems. Given the limitations intrinsic to all retrospective studies, the findings of the risk factor analysis should be interpreted within the limitations of its study design, such as non-standardized follow-up time points, incomplete data/attrition, and variation in follow-up documentation. Because we did not include any comparison/control groups, we can only report our observations and refrain from making definitive claims about treatment or technique superiority/inferiority. Moreover, the PRO changes may be confounded by other aspects of patients' medical management, including psychological support, home exercises, and over-the-counter oral analgesics. Lastly, the findings from this cohort at a large academic medical center are likely more generalizable to certain metropolitan populations but less applicable to rural populations.

## Conclusions

GNRFA has been demonstrated as an effective option in the treatment course for chronic knee pain and symptomatic knee OA. In this real-world cohort, conventional GNRFA using the classical three-site technique demonstrated robust short-term pain reduction, and at least half of the cohort maintained clinically meaningful pain improvements for 11 months. For chronic knee pain, GNRFA is a reasonable therapeutic option in appropriately selected patients.

## References

[1] Conger A, Gililland J, Anderson L, Pelt CE, Peters C, McCormick ZL (2021). Genicular nerve radiofrequency ablation for the treatment of painful knee osteoarthritis: current evidence and future directions. Pain Med.

[2] Belba A, Vanneste T, Vanlinthout LE (2025). Radiofrequency treatment for chronic knee pain in people with knee osteoarthritis. Cochrane Database Syst Rev.

[3] Tran J, Peng PW, Lam K, Baig E, Agur AM, Gofeld M (2018). Anatomical study of the innervation of anterior knee joint capsule: implication for image-guided intervention. Reg Anesth Pain Med.

[4] Franco CD, Buvanendran A, Petersohn JD, Menzies RD, Menzies LP (2015). Innervation of the anterior capsule of the human knee: implications for radiofrequency ablation. Reg Anesth Pain Med.

[5] Choi WJ, Hwang SJ, Song JG, Leem JG, Kang YU, Park PH, Shin JW (2011). Radiofrequency treatment relieves chronic knee osteoarthritis pain: a double-blind randomized controlled trial. Pain.

[6] Yasar E, Kesikburun S, Kılıç C, Güzelküçük Ü, Yazar F, Tan AK (2015). Accuracy of ultrasound-guided genicular nerve block: a cadaveric study. Pain Physician.

[7] Abd-Elsayed A, Strand N, Gritsenko K, Martens J, Chakravarthy K, Sayed D, Deer T (2022). Radiofrequency ablation for the knee joint: a survey by the American Society of Pain and Neuroscience. J Pain Res.

[8] Conger A, Cushman DM, Walker K, Petersen R, Walega DR, Kendall R, McCormick ZL (2019). A novel technical protocol for improved capture of the genicular nerves by radiofrequency ablation. Pain Med.

[9] Koshi E, Cheney CW, Sperry BP, Conger A, McCormick ZL (2020). Genicular nerve radiofrequency ablation for chronic knee pain using a three-tined electrode: a technical description and case series. Pain Med.

[10] Chen AF, Khalouf F, Zora K (2020). Cooled radiofrequency ablation compared with a single injection of hyaluronic acid for chronic knee pain: a multicenter, randomized clinical trial demonstrating greater efficacy and equivalent safety for cooled radiofrequency ablation. J Bone Joint Surg Am.

[11] Wong PK, Kokabi N, Guo Y, Reiter D, Reimer NB, Oskouei S, Gonzalez FM (2021). Safety and efficacy comparison of three- vs four-needle technique in the management of moderate to severe osteoarthritis of the knee using cooled radiofrequency ablation. Skeletal Radiol.

[12] Sperry BP, Conger A, Kohan L, Walega DR, Cohen SP, McCormick ZL (2021). A proposed protocol for safe radiofrequency ablation of the recurrent fibular nerve for the treatment of chronic anterior inferolateral knee pain. Pain Med.

[13] Cohen SP, Gerdesmeyer L, McCormick ZL (2024). Non-surgical interventional treatments for knee osteoarthritis: the need for different perspectives, cross-specialty collaboration, and preclinical and translational research. Interv Pain Med.

[14] Caragea M, Woodworth T, Curtis T (2023). Genicular nerve radiofrequency ablation for the treatment of chronic knee joint pain: a real-world cohort study with evaluation of prognostic factors. Pain Med.

[15] Nnake CO, El-Othmani MM, Cooper HJ, Shah RP, Geller JA, Neuwirth AL (2024). Genicular nerve radiofrequency ablation: a systematic review of application for perioperative pain control in total knee arthroplasty and as treatment for chronic pain in well-appearing total knee arthroplasty. Knee Surg Relat Res.

[16] Shi W, Vu TN, Annaswamy T (2024). Effectiveness comparison of genicular nerve ablation for knee osteoarthritic versus post-total knee arthroplasty pain. Interv Pain Med.

[17] Brown TK, Caragea MA, Beckwith M (2025). Evaluating the effectiveness of genicular radiofrequency ablation for chronic knee pain using the Patient-Reported Outcomes Measurement Information System (PROMIS) Global Health-Physical Health domain: results of a cross-sectional cohort study. Interv Pain Med.

[18] Kellgren JH, Lawrence JS (1957). Radiological assessment of osteo-arthrosis. Ann Rheum Dis.

[19] Kohn MD, Sassoon AA, Fernando ND (2016). Classifications in brief: Kellgren-Lawrence classification of osteoarthritis. Clin Orthop Relat Res.

[20] Cella D, Riley W, Stone A (2010). The Patient-Reported Outcomes Measurement Information System (PROMIS) developed and tested its first wave of adult self-reported health outcome item banks: 2005-2008. J Clin Epidemiol.

[21] Löwe B, Unützer J, Callahan CM, Perkins AJ, Kroenke K (2004). Monitoring depression treatment outcomes with the Patient Health Questionnaire-9. Med Care.

[22] Farrar JT, Young JP Jr, LaMoreaux L, Werth JL, Poole MR (2001). Clinical importance of changes in chronic pain intensity measured on an 11-point numerical pain rating scale. Pain.

[23] Salaffi F, Stancati A, Silvestri CA, Ciapetti A, Grassi W (2004). Minimal clinically important changes in chronic musculoskeletal pain intensity measured on a numerical rating scale. Eur J Pain.

[24] Braun S, Mascoe J, Caragea M (2024). The association of payer type on genicular radiofrequency neurotomy treatment outcomes: results of a cross-sectional study. Interv Pain Med.

[25] Kirdemir P, Çatav S, Alkaya Solmaz F (2017). The genicular nerve: radiofrequency lesion application for chronic knee pain. Turk J Med Sci.

[26] Santana Pineda MM, Vanlinthout LE, Moreno Martín A, van Zundert J, Rodriguez Huertas F, Novalbos Ruiz JP (2017). Analgesic effect and functional improvement caused by radiofrequency treatment of genicular nerves in patients with advanced osteoarthritis of the knee until 1 year following treatment. Reg Anesth Pain Med.

[27] McCormick ZL, Reddy R, Korn M (2018). A prospective randomized trial of prognostic genicular nerve blocks to determine the predictive value for the outcome of cooled radiofrequency ablation for chronic knee pain due to osteoarthritis. Pain Med.

[28] Fogarty AE, Burnham T, Kuo K (2022). The effectiveness of fluoroscopically guided genicular nerve radiofrequency ablation for the treatment of chronic knee pain due to osteoarthritis: a systematic review. Am J Phys Med Rehabil.

[29] Ikeuchi M, Ushida T, Izumi M, Tani T (2011). Percutaneous radiofrequency treatment for refractory anteromedial pain of osteoarthritic knees. Pain Med.

[30] Wu BP, Grits D, Foorsov V, Xu J, Tankha P, Bolash RB (2022). Cooled and traditional thermal radiofrequency ablation of genicular nerves in patients with chronic knee pain: a comparative outcomes analysis. Reg Anesth Pain Med.

[31] Li G, Zhang Y, Tian L, Pan J (2021). Radiofrequency ablation reduces pain for knee osteoarthritis: a meta-analysis of randomized controlled trials. Int J Surg.

[32] Malaithong W, Tontisirin N, Seangrung R, Wongsak S, Cohen SP (2022). Bipolar radiofrequency ablation of the superomedial (SM), superolateral (SL) and inferomedial (IM) genicular nerves for chronic osteoarthritis knee pain: a randomized double-blind placebo-controlled trial with 12-month follow-up. Reg Anesth Pain Med.

[33] El-Hakeim EH, Elawamy A, Kamel EZ (2018). Fluoroscopic guided radiofrequency of genicular nerves for pain alleviation in chronic knee osteoarthritis: a single-blind randomized controlled trial. Pain Physician.

[34] Hashemi M, Nabi BN, Saberi A, Sedighinejad A, Haghighi M, Farzi F, Biazar G (2016). The comparison between two methods for the relief of knee osteoarthritis pain: radiofrequency and intra-periarticular ozone injection: a clinical trial study. Int J Med Res Health Sci.

[35] Ma Y, Chen YS, Liu B, Sima L (2024). Ultrasound-guided radiofrequency ablation for chronic osteoarthritis knee pain in the elderly: a randomized controlled trial. Pain Physician.

[36] Belba A, Vanneste T, Kallewaard JW (2025). Cooled versus conventional radiofrequency treatment of the genicular nerves for chronic knee pain: 12-month and cost-effectiveness results from the multicenter COCOGEN trial. Reg Anesth Pain Med.

[37] Kanjanapanang N, Madrid R, Lin P (2026). Effectiveness of genicular nerve radiofrequency ablation in osteoarthritis and post-surgical knee pain: systematic review. Pain Med.

[38] Kwon HJ, Kim CS, Kim DH, Shin JW, Choi D, Choi SS (2024). Effectiveness of the cooled radiofrequency ablation of genicular nerves in patients with chronic knee pain due to osteoarthritis: a double-blind, randomized, controlled study. Medicina (Kaunas).

[39] McCormick ZL, Korn M, Reddy R (2017). Cooled radiofrequency ablation of the genicular nerves for chronic pain due to knee osteoarthritis: six-month outcomes. Pain Med.

[40] Hunter C, Davis T, Loudermilk E, Kapural L, DePalma M (2020). Cooled radiofrequency ablation treatment of the genicular nerves in the treatment of osteoarthritic knee pain: 18- and 24-month results. Pain Pract.

[41] Lyman J, Khalouf F, Zora K (2022). Cooled radiofrequency ablation of genicular nerves provides 24-month durability in the management of osteoarthritic knee pain: outcomes from a prospective, multicenter, randomized trial. Pain Pract.

[42] Chau J, Potu C, Anesi T, Stephens A (2026). Rates of knee arthroplasty after genicular nerve ablation: a retrospective study utilizing a large national database. Interv Pain Med.

[43] Phyomaung PP, Dubowitz J, Cicuttini FM (2014). Are depression, anxiety and poor mental health risk factors for knee pain? A systematic review. BMC Musculoskelet Disord.

[44] Lewis GN, Rice DA, McNair PJ, Kluger M (2015). Predictors of persistent pain after total knee arthroplasty: a systematic review and meta-analysis. Br J Anaesth.

[45] Kose SG, Kose HC, Celikel F, Akkaya OT (2022). Predictive factors associated with successful response to utrasound guided genicular radiofrequency ablation. Korean J Pain.

[46] Philip A, Williams M, Davis J (2021). Evaluating predictors of pain reduction after genicular nerve radiofrequency ablation for chronic knee pain. Pain Manag.

[47] Flor H (2014). Psychological pain interventions and neurophysiology: implications for a mechanism-based approach. Am Psychol.

[48] Hobelmann JG, Huhn AS (2021). Comprehensive pain management as a frontline treatment to address the opioid crisis. Brain Behav.

